# Deep brain stimulation and magnetic resonance–guided focused ultrasound for essential tremor: a meta-analysis of effectiveness and safety

**DOI:** 10.1007/s10143-025-04076-x

**Published:** 2026-03-09

**Authors:** Haneen Sabet, Abdallah Abbas, Esraa Y. Salama, Abrar AbuHamdia, Mohamed El-Moslemani, Majed Aldehri, Ibrahim Alnaami

**Affiliations:** 1 Faculty of Medicine, South Valley University, Qena, Egypt; 2 Faculty of Medicine, Al-Azhar University, Damietta, Egypt; 3 Faculty of Medicine, Benha University, Benha, Egypt; 4 Neurological Disorders Research Center, Qatar Biomedical Research Institute, Hamad Bin Khalifa University, Qatar Foundation, Education City, 34110 Doha, Qatar; 5 Department of Anatomy, College of Medicine, King Khalid University, 808262523 Abha, Saudi Arabia; 6 Department of Neurosurgery, Mental Health and Neuroscience, Maastricht University Medical Center, Maastricht, Netherlands; 7 Division of Neurosurgery, Department of Surgery, King Khalid University, Abha, Saudi Arabia

**Keywords:** Deep brain stimulation, DBS, Essential tremor, ET, Magnetic resonance–guided focused ultrasound, MRgFUS

## Abstract

We compared the effectiveness and safety of deep brain stimulation (DBS) and magnetic resonance-guided focused ultrasound (MRgFUS) in patients with essential tremor (ET). A comprehensive search was conducted across four databases (Web of Science, Scopus, PubMed, and Cochrane CENTRAL) till June 2025. Clinical trials and observational studies comparing DBS and MRgFUS in patients with ET were included. Meta-analysis was performed using RevMan version 5.4 and OpenMetaAnalyst, applying a random-effect model. Five cohort studies (463 patients) were included. In the total Clinical Rating Scale for Tremor (CRST) score, there was a significant difference between the two groups favoring DBS (mean difference (MD): 5.12, 95% confidence interval (CI): [1.08, 9.16], *P* = 0.01), while in part A of the CRST, there was no significant difference (*P* = 0.15). No significant difference was found between the two groups in response rate as well (*P* = 0.56). Paresthesia (13.3% vs. 2.2%; risk ratio (RR): 4.57, 95% CI: [1.31, 15.94], *P* = 0.02), gait instability (26.56% vs. 4.06%; RR: 2.85, 95% CI: [1.14, 7.08], *P* = 0.025), and dysphagia (7.77% vs. 1.37%; RR: 3.40, 95% CI: [1.01, 11.46], *P* = 0.049) were significantly more frequent in the FUS group compared with DBS. Other serious but rare adverse events (AEs), such as infection and hemorrhage, were more common in the DBS group, but none significantly differed between the two groups. Both DBS and MRgFUS showed similar response rates; however, DBS may provide greater improvement in the total CRST score, potentially due to the comparison of bilateral DBS against predominantly unilateral MRgFUS. Regarding safety, MRgFUS was more associated with sensory and motor AEs, while infrequent but potentially serious complications were only reported with DBS. Future high-quality prospective studies with larger sample sizes and longer follow-up periods are needed.

**Supplementary Information:**

The online version contains supplementary material available at 10.1007/s10143-025-04076-x.

## Introduction

Essential tremor (ET) is the most prevalent movement disorder, affecting approximately 3.2 individuals per 1,000 globally. Characterized primarily by an isolated upper limb action tremor persisting for at least three years, ET may also present with tremors in other regions [1]. Management strategies for ET primarily involve pharmacological and surgical interventions. Propranolol is the only U.S. Food and Drug Administration (FDA)-approved medication for ET. For patients resistant to medical management, surgical options are often required [1].

Deep brain stimulation (DBS) is a well-established, reversible method to alleviate ET through targeted electrical stimulation of the ventral intermediate nucleus (Vim) of the thalamus. While DBS offered substantial benefits, including adjustable stimulation, it requires invasive procedures and the implantation of hardware [2–5]. Conversely, magnetic resonance-guided focused ultrasound (MRgFUS) has emerged as a non-invasive alternative. It leverages focused ultrasound (FUS) energy to create precise lesions in the brain without incisions [6]. This technique combines the accuracy of real-time magnetic resonance imaging (MRI) with the therapeutic effects of ultrasound and has been recently approved for the treatment of ET [5, 7–9].

Despite the distinct mechanisms of these two modalities, current literature suggests comparable effectiveness and safety, yet a systematic review and meta-analysis that thoroughly examines and directly compares these studies is lacking. This creates a critical knowledge gap regarding the optimal surgical choice for medication-refractory patients. This systematic review and meta-analysis aim to fill this gap by rigorously comparing the effectiveness and safety of DBS and MRgFUS in treating patients with ET.

## Methods

This study followed the guidelines outlined in the Cochrane Handbook for Systematic Reviews of Interventions, the PRISMA statement [10, 11], and the MOOSE guidelines for meta-analyses of observational studies [12]. The protocol was registered in the PROSPERO (CRD420251158889).

### Search & eligibility criteria

We searched four databases—Web of Science, Scopus, PubMed, and Cochrane CENTRAL—from their inception until June 2025. The search query used was:

(("Deep brain stimulation" OR DBS) AND ("Focused ultrasound" OR FUS)) AND ("Essential tremor" OR "Essential tremors" OR "Kinetic tremor" OR "Kinetic tremors" OR "Familial tremor" OR "Familial tremors"). For the detailed search strategy across each database, refer to Supplementary Table 1.

We included all clinical trials and observational studies (both retrospective and prospective) that compared two groups—one receiving DBS and the other FUS—in patients with ET. The studies had to aim at evaluating the efficacy and safety of these two treatment modalities. There was no language restriction.

We excluded studies with different designs (case series, abstracts, animal studies, reviews, and case reports), as well as studies involving different populations (e.g., patients with PD), single-arm studies, or studies with outcomes unrelated to effectiveness or safety.

### Screening & data extraction

The screening process was conducted in two steps: first by reviewing titles and abstracts, followed by full-text screening. Two independent and blinded authors carried out the process using the Rayyan tool [13].

We applied the same approach for data extraction. A spreadsheet was used to collect the following information: summary data, baseline data, and outcomes. The summary data included study design, recruitment duration, follow-up period, country, details of FUS and DBS (whether unilateral or bilateral, target, and imaging modality), inclusion criteria, and a general summary of the study. Baseline data included the sample size (number of patients), age (in years, mean and standard deviation [SD]), sex (number of males and females), disease duration (in years, mean and SD), baseline Clinical Rating Scale for Tremor [CRST] score (mean and SD), and baseline quality of life in essential tremor questionnaire [QUEST] score (mean and SD).

Outcome data included the response rate, CRST scores, and adverse events (AEs). The response rate was defined differently in two studies. In Kim et al. (2017), it was defined as complete tremor remission after surgery or a reduction in tremor greater than 90% [14]. In Huss et al. (2015), it was defined as a reduction in dominant hand action or intention tremor on the CRST score from a rating of 2 to 4 to a rating of 0 to 1 [15].

The CRST included Part A and the total score. The CRST is a tool used to assess the severity of tremor symptoms and their impact on daily life [16]. It consists of three parts. Part A assesses tremor severity at rest, in posture, and during movement (intention tremor). Part B evaluates how tremor affects motor tasks such as writing, drawing, and pouring water. Part C assesses the functional impact of tremor on daily activities such as eating, dressing, and using utensils. Each item on the scale is typically scored from 0 to 4, with 0 indicating no tremor and higher scores reflecting more severe symptoms. The total CRST score is the sum of all three parts, with higher scores indicating greater severity and functional impairment.

### Bias risk assessment

We used the Newcastle–Ottawa Scale (NOS) tool to assess the bias risk in retrospective observational studies. For details about the domain and scoring system, refer to Supplementary Table 2.

### Meta-analysis 

The meta-analysis was performed using Review Manager (RevMan) version 5.4 or OpenMetaAnalyst [17, 18]. We used OpenMetaAnalyst in cases where the event count was zero in both the DBS and FUS groups, applying a correction factor of 0.5, as recommended by the Cochrane Handbook when both arms include zero events [19], since this function is not available in RevMan. For continuous outcomes, we analyzed the pooled mean difference (MD) with a 95% confidence interval (CI). We opted for the MD over the Standardized Mean Difference (SMD) because all included studies utilized the same assessment tool (CRST) and reported scores in the same units (points). This allowed for a direct, clinically meaningful comparison of the absolute treatment effect between DBS and MRgFUS. For dichotomous outcomes, such as the incidence of AEs and response rate, we calculated the Risk Ratio (RR) and its 95% CI.

We applied a random-effects model because it better accounts for heterogeneity or variability between groups or individuals [20]. In random-effects models, smaller studies are given relatively more weight compared to fixed-effect models. This model also allows for greater variability in the pooled estimate, which is more suitable for data that may contain inconsistencies or variations. As a result, the effect sizes derived from our meta-analysis are conservative estimates that take possible heterogeneity into consideration.

Heterogeneity was considered significant if the *p*-value was less than 0.1 or the I^2^ statistic was greater than 50% [21]. When significant heterogeneity was present, we performed a leave-one-out analysis to address it [22]. Since fewer than ten studies were included, assessment of publication bias was not applicable, in accordance with Cochrane guidelines [23].

## Results

### Search and screening

**Fig. 1:**
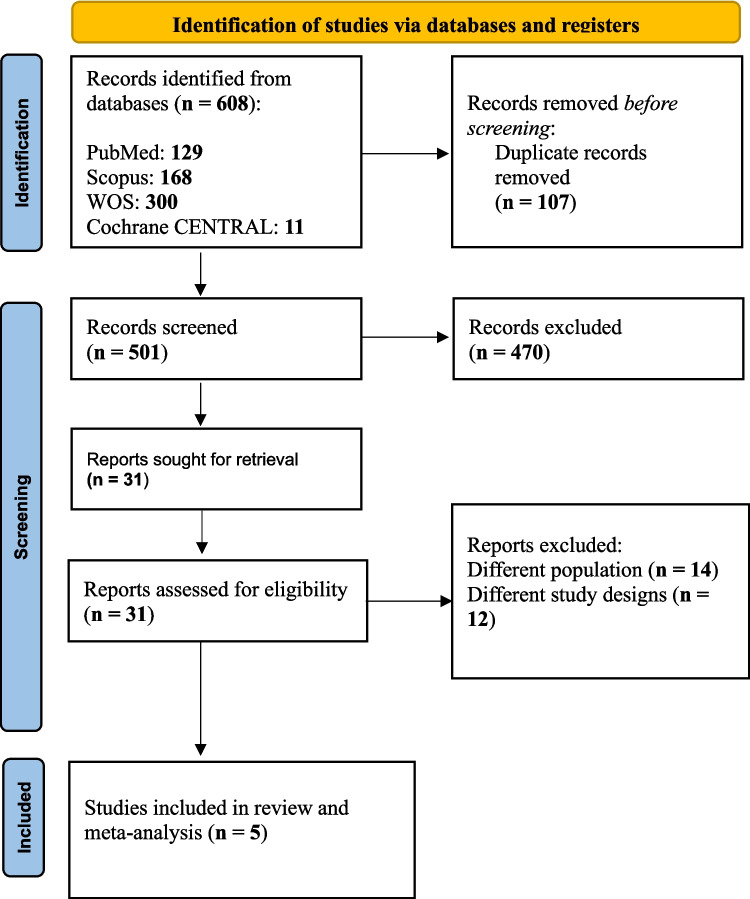
PRISMA flow diagram of study selection. Flow chart illustrating the process of study identification, screening, eligibility assessment, and final inclusion. A total of 608 records were identified, with 5 studies meeting the inclusion criteria for the meta-analysis

Our initial database search retrieved 608 studies, then we removed the duplicates to have 501 articles ready for title and abstract screening. We excluded 470 articles for not meeting the eligibility criteria, resulting in the inclusion of 31 articles for the full-text screening. Ultimately, five studies [14, 15, 24–26] remained for data extraction and analysis as thoroughly described in Fig. 1.

### Summary and baseline characteristics

Table 1 shows five retrospective cohort studies (2 in the USA, 2 in Canada, and 1 in South Korea), with a mean follow-up period of 15 months. All studies in the FUS reported unilateral treatment targeting the Vim using MRI guidance, whereas the DBS reported bilateral and unilateral procedures targeting the Vim. All the studies in the DBS group used MRI guidance except the Huss et al. [15] study, which was the only study to report computed tomography (CT) and 1.5- or 3-Tesla (T) MRI in the imaging modality.

**Table 1:** Summary of included studies

Study ID	Study design	Recruitment duration	Follow-up	Country	FUS details			DBS details			Inclusion criteria	Summary of the study
					Unilateral/Bilateral	Target	Imaging	Unilateral/Bilateral	Target	Imaging		
Huss 2015	Retrospective cohort	2004–2013	13 months	USA	Unilateral	VIM	MRI	Both	VIM	CT and 1.5 or 3-T MRI	Patients with ET underwent both preoperative and postoperative CRST evaluations by the same neurologic specialist and physical therapist	The study showed that bilateral DBS is more effective than unilateral DBS or FUS in improving tremors
Kim 2017	Retrospective cohort	1995–2014	12 months	South Korea	Unilateral	VIM	3-T MRI	Both	VIM	1.5-T MRI	Patients with drug-resistant ET	Bilateral thalamic DBS reduces overall tremor more than unilateral DBS or FUS; however, both unilateral and bilateral treatments are equally effective for contralateral hand tremor and show no significant differences in disability or quality of life outcomes
Harary 2019	Retrospective cohort	NA	12 months	USA	Unilateral	VIM	MRI	Both	VIM	MRI	Patients with ET	Patients with drug-resistant ET experienced similar outcomes from RF thalamotomy, DBS, and MRgFUS, with fewer treatment-related complications observed in DBS and MRgFUS
Germann 2024	Retrospective cohort	NA	13.8 months	Canada	Unilateral	VIM	1.5–3 T MRI	Unilateral	VIM	1.5–3 T MRI	Patients with ET who have hand tremor that is medically refractory to two full doses of therapeutic medication and who have substantial disability in performing at least two daily activities	Comparison of DBS and MRgFUS efficacy maps suggests a potentially more antero-superior lesion target that may reduce complications while preserving efficacy
Sarica 2025	Retrospective cohort	2000–2024	24.5 months	Canada	Bilateral	VIM	MRI	Bilateral	VIM	MRI	Patients with ET who underwent successful first-side surgery and exhibited residual tremors on the contralateral side	Bilateral MRgFUS and DBS seem equally effective

*CRST* clinical rating scale for tremor, *CT* computed tomography, *DBS* deep brain stimulation, *ET* essential tremor, *FUS* focused ultrasound, *MRI* magnetic resonance imaging, *MRgFUS* magnetic resonance-guided focused ultrasound, *RF* radiofrequency, *T* Tesla, *VIM* ventral intermediate nucleus

Table 2 summarizes the baseline characteristics of 192 patients (133 males, 59 females) in the FUS group and 271 patients (173 males, 98 females) in the DBS group. The pooled mean age and the SD in the FUS group and DBS group were 66.8 ± 14.8 and 62.3 ± 13.9 years, respectively. Moreover, the pooled mean of the disease duration is 30.7 years with an SD of 17.7 in the FUS group, compared to a pooled mean of 27.5 years with an SD of 17.3 in the DBS group. The baseline CRST in the FUS group has a pooled mean of 57.4 with an SD of 10.4, compared to a pooled mean of 58.7 with an SD of 11.2 in the DBS group.

**Table 2:** Baseline characteristics of patients in FUS and DBS groups

Study ID	Sample size (no.)		Age, years, mean (SD)		Sex, Males No: Females No		Disease duration (y), mean (SD)		Baseline total CRST, mean (SD)		Baseline QUEST, mean (SD)	
	FUS	DBS	FUS	DBS	FUS	DBS	FUS	DBS	FUS	DBS	FUS	DBS
Huss 2015	15	70	67.2	65.02 (11.9)	10:5	58:12	NR	NR	54.9	61.9	37.5	52.1
Kim 2017	23	19	64.7 (8.04)	62.8 (13.5)	20:3	13:6	20.5 (13.13)	14.1 (7.317)	NR	NR	NR	NR
Harary 2019	56	127	70.8 (8.7)	64.6 (9.6)	37:19	69:58	28.3 (16.4)	29.1 (17.4)	NR	NR	42.6	49.1
Germann 2024	79	33	71.1 (9)	65.6 (9.4)	53:26	22:11	35.4 (18.4)	28.8 (18.1)	57.7 (15.8)	58.7 (11.03)	NR	NR
Sarica 2025	19	22	35.5 (17.9)	34.9 (18.2)	13:6	11:11	NR	NR	56.3 (7.6)	58.8 (11.7)	NR	NR

*CRST* clinical rating scale for tremor, *DBS* deep brain stimulation, *FUS* focused ultrasound, *M* males, *F* females, *NR* not reported, *QUEST* quality of life in essential tremor questionnaire, *SD* standard deviation, *y* years

### Risk of bias assessment

The NOS tool evaluated the five included retrospective cohorts. Four studies [14, 15, 24, 25] showed good quality and scored 8 out of 9. However, one study [26] showed poor quality and scored 6 out of 9 due to the lack of comparability (see Supplementary Table 2).

### Clinical rating scale for tremor (CRST)

**Fig. 2:**
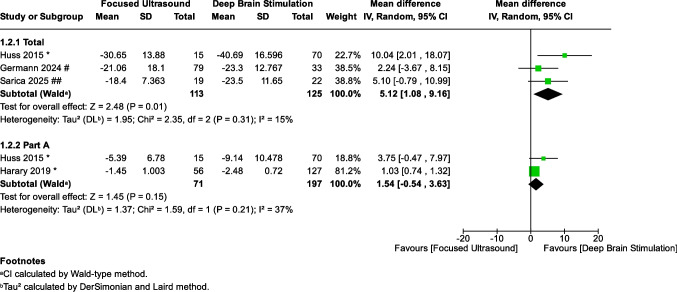
Forest plot of the effect of DBS vs. FUS on total and part A CRST scores. Meta-analysis comparing the change from baseline in total CRST and CRST Part A scores between patients undergoing DBS and those receiving FUS. DBS showed significantly greater improvement in total CRST (MD: 5.12, 95% CI: [1.08, 9.16], *P* = 0.01), with insignificant heterogeneity (*I*^2^ = 15%, *P* = 0.31). No significant difference was found in CRST Part A (MD: 1.54, 95% CI: [−0.54, 3.63], *P* = 0.15), with insignificant heterogeneity (*I*^2^ = 37%, *P* = 0.21). (*: 12 months, #: 41 months, ##: 16 months)

Four studies [15, 24–26], including 421 patients (169 in FUS and 252 in DBS), reported CRST outcomes. Analysis of the change from baseline in total CRST score favored DBS over FUS, showing a statistically significant greater improvement for DBS (MD: 5.12, 95% CI: [1.08, 9.16], *P* = 0.01) (Fig. 2). Heterogeneity was insignificant across the studies (*I*^2^ = 15%, *P* = 0.31). In contrast, there was no statistically significant difference between the FUS and DBS groups regarding the change in CRST Part A score (MD: 1.54, 95% CI: [−0.54, 3.63], *P* = 0.15), with insignificant heterogeneity (*I*^2^ = 37%, *P* = 0.21) (Fig. 2).

### Response rate

**Fig. 3:**
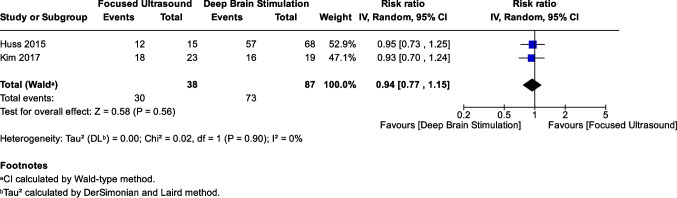
Forest plot of the response rate between DBS and FUS groups. Pooled analysis of the response rate at 12 months among patients undergoing DBS and FUS. No statistically significant difference was observed between the two groups (RR: 0.94, 95% CI: [0.77, 1.15], *P* = 0.56), with no heterogeneity (*I*^2^ = 0%, *P* = 0.90)

The 12-month response rate was reported in two studies with 125 patients (38 in FUS and 87 in DBS) [14, 15]. There was no statistically significant difference in the response rate between the two groups (RR: 0.94, 95% CI: [0.77, 1.15], *P* = 0.56) (Fig. 3). The pooled results were homogenous (*I*^2^ = 0%, *P* = 0.90).

### Adverse events (AEs)

**Fig. 4:**
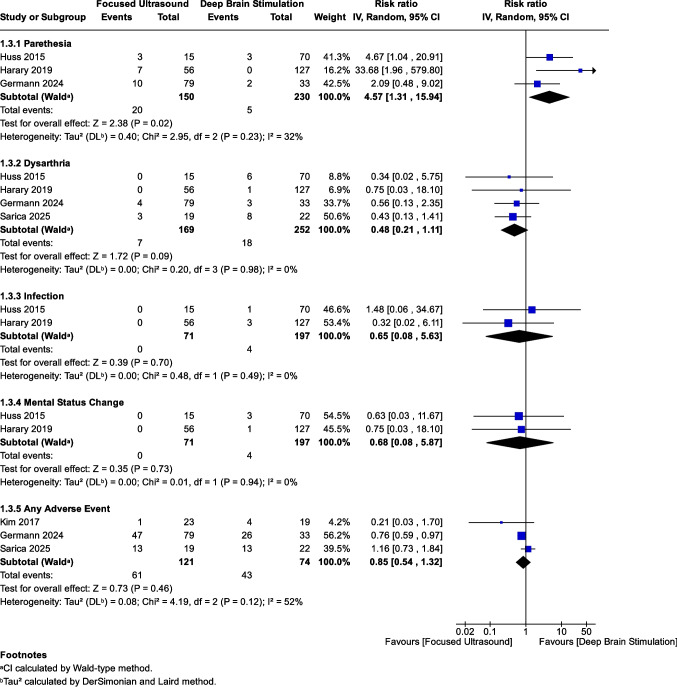
Forest plot of adverse events in DBS vs. FUS. Meta-analysis comparing the incidence of multiple adverse events between patients treated with DBS and FUS. Paresthesia was significantly more common in the FUS group (RR: 4.57, 95% CI: [1.31, 15.94], *P* = 0.02), with insignificant heterogeneity (*I*^2^ = 32%, *P* = 0.23). There was no significant difference between the groups in the risk of dysarthria (RR: 0.48, 95% CI: [0.21, 1.11], *P* = 0.09; *I*^2^ = 0%, *P* = 0.98), infection (RR: 0.65, 95% CI: [0.08, 5.63], *P* = 0.70; *I*^2^ = 0%, *P* = 0.49), or mental status changes (RR: 0.68, 95% CI: [0.08, 5.87], *P* = 0.73; *I*^2^ = 0%, *P* = 0.94). The pooled analysis of any adverse event showed no statistically significant difference between FUS and DBS (RR: 0.85, 95% CI: [0.54, 1.32], *P* = 0.46), with moderate heterogeneity (*I*^2^ = 52%, *P* = 0.12)

**Fig. 5:**
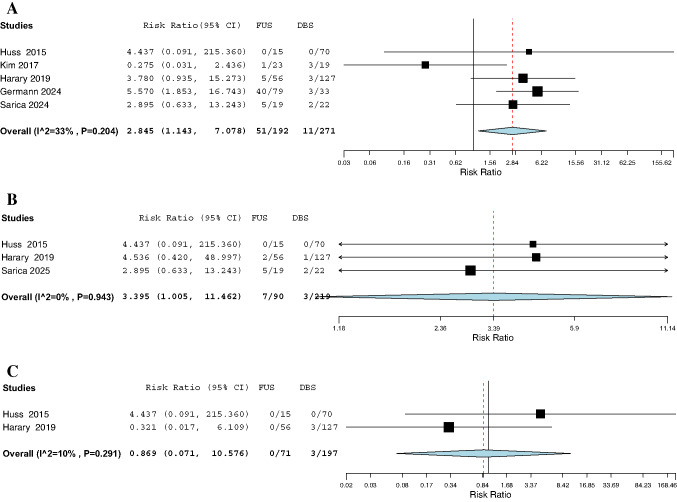
**A** Forest plot of gait instability in DBS vs. FUS. Patients undergoing FUS had a significantly higher risk of gait instability compared to those treated with DBS (RR: 2.85, 95% CI: [1.14, 7.08], *P* = 0.025), with insignificant heterogeneity (*I*^2^ = 33%, *P* = 0.204). **B** Forest plot of dysphagia in DBS vs. FUS. Pooled analysis revealed a higher incidence of dysphagia in the FUS group compared to DBS (RR: 3.40, 95% CI: [1.01, 11.46], *P* = 0.049), with no heterogeneity (*I*^2^ = 0%, *P* = 0.943). **C** Forest plot of hemorrhage in DBS vs. FUS. No statistically significant difference in the risk of hemorrhage was found between FUS and DBS (RR: 0.87, 95% CI: [0.07, 10.58], *P* = 0.91), with insignificant heterogeneity (*I*^2^ = 10%, *P* = 0.291)

We analyzed AEs reported in at least two studies; all AEs are reported in Supplementary Table 3. The overall incidence of any AE did not differ significantly between FUS (50.4%) and DBS (58.1%) (RR: 0.85, *P* = 0.46), as shown in Fig. 4. Paresthesia (RR: 4.57, *P* = 0.02), gait instability (RR: 2.85, *P* = 0.025), and dysphagia (RR: 3.40, *P* = 0.049) were all significantly more frequent in the FUS group. Specifically, gait instability is shown in Fig. 5A and dysphagia in Fig. 5B. Rates of dysarthria (RR: 0.48, *P* = 0.09), infection (RR: 0.65, *P* = 0.70), and mental status changes (RR: 0.68, *P* = 0.73) did not differ significantly between FUS and DBS (Fig. 4). Similarly, there was no significant difference in the risk of hemorrhage (RR: 0.87, *P* = 0.913), as shown in Fig. 5C.

## Discussion

Our meta-analysis demonstrated that DBS provides significantly greater improvement in total CRST scores compared with MRgFUS, whereas no significant differences were observed for CRST Part A or the 12-month response rate. FUS was associated with higher rates of paresthesia, gait instability, and dysphagia, while other AEs, including dysarthria, infection, hemorrhage, and mental status changes, were comparable between groups. Overall, these findings reflect a trade-off in which DBS offers stronger total tremor suppression, whereas MRgFUS carries a higher likelihood of certain sensory and gait-related AEs.

Both DBS and MRgFUS modulate tremor circuitry at the level of the Vim, a major relay within the cerebello–thalamo–cortical network implicated in ET. DBS achieves tremor reduction by applying adjustable electrical stimulation that modulates pathological oscillations within this circuit, allowing postoperative titration and management of stimulation-related side effects. In contrast, MRgFUS produces a precise, MRI-guided thermal lesion that permanently disrupts the aberrant tremor pathway [27–29]. The irreversibility of MRgFUS underscores the importance of accurate targeting, whereas DBS offers ongoing flexibility that may contribute to its overall efficacy profile.

Regarding study quality, most included cohorts were of good methodological quality, although one study had limited comparability [26]. Sensitivity analyses excluding this study showed that the direction of effect for most outcomes remained unchanged, supporting the robustness of the primary findings.

Interpretation of laterality is essential when comparing modalities. DBS was frequently performed bilaterally in the included studies, whereas MRgFUS was predominantly unilateral [14, 15, 25, 26]. This likely contributed to the superior total CRST improvement observed with DBS. Patients undergoing staged bilateral DBS often demonstrate strong first-side outcomes and fewer AEs before receiving the second implant, introducing a degree of selection bias. Conversely, evidence for bilateral MRgFUS remains extremely limited; only one short-term study has reported outcomes, and its highly selective design limits broader applicability [24].

The knowledge gap surrounding bilateral MRgFUS represents one of the most critical limitations in the current literature. Although long-term data for bilateral DBS demonstrate a gradual attenuation of benefit, such as the decline from 57 to 43% improvement over four years reported by Sarica et al. [24], no comparable long-term data exist for bilateral MRgFUS. Furthermore, the long-term safety of bilateral thermal lesions, particularly with respect to gait, speech, and sensory function, remains unknown. This lack of data prevents definitive comparisons between bilateral MRgFUS and its DBS counterpart and should be highlighted as a central limitation.

Differences in AE profiles between procedures also warrant clinical consideration. MRgFUS carries a higher incidence of sensory and gait-related complications, likely due to perilesional edema or lesion extension into adjacent fiber tracts [26]. Neurological AEs may decline as operator experience increases, yet current data remain heterogeneous. DBS, on the other hand, presents a distinct set of risks, such as infection, hemorrhage, or hardware-related complications, that were infrequent but exclusive to the DBS cohorts. The adjustability of DBS allows many stimulation-related AEs to be mitigated postoperatively [24], which may contribute to its overall safety profile in long-term use.

Targeting considerations further differentiate both approaches. The irreversibility of MRgFUS makes optimal lesion placement crucial, whereas DBS enables postoperative refinement through contact selection and stimulation adjustments. Evidence from recent connectomic and probabilistic mapping studies suggests that networks involving the primary motor cortex (M1), primary somatosensory cortex (S1), cerebellum, and supplementary motor area play key roles in tremor suppression [30, 31]. How these findings translate to optimizing MRgFUS targeting remains an area requiring further investigation.

Clinically, bilateral approaches appear most appropriate for patients with significant axial tremor and functional impairment, given the substantial improvements observed after second-side DBS. Unilateral procedures may suffice for patients with predominantly asymmetric tremor. Treatment selection should therefore integrate tremor laterality, patient comorbidities, willingness to manage implanted hardware, and tolerance for sensory or gait-related risks.

Overall, the current evidence indicates that DBS may provide greater total tremor reduction, whereas MRgFUS offers a non-invasive alternative with a distinct AE profile. However, the absence of long-term and bilateral MRgFUS data remains a substantial limitation that restricts definitive comparisons. Future prospective studies with balanced bilateral designs, longer follow-up intervals, and standardized reporting will be essential to refining patient selection and optimizing therapeutic outcomes.

### Limitations and recommendations

Our systematic review and meta-analysis are subject to several limitations. The small number of included studies, with several key outcomes, including response rate, CRST Part A, and numerous individual AEs (e.g., mental status changes, infection, and hemorrhage), reported in only two studies, limits the generalizability and robustness of conclusions that can be drawn from these specific outcomes. Additionally, only one study reported outcomes for bilateral MRgFUS, preventing subgroup analyses based on procedure laterality. While four of five studies displayed a low risk of bias, one study revealed poor quality due to issues with comparability. The overall sample size is relatively small, with a higher percentage of male participants: 192 patients (69.27% male) in the MRgFUS group and 271 patients (63.84% male) in the DBS group. Most studies primarily compared bilateral DBS with unilateral MRgFUS, which may introduce bias given the recognized advantages of bilateral approaches. This focus could lead to an overestimation of DBS's benefits when compared to MRgFUS. Additionally, selection bias may influence findings, as patients with more severe conditions are often referred for DBS, potentially skewing the results. Those undergoing bilateral procedures are likely to demonstrate better first-side outcomes and experience fewer AEs, complicating assessments of efficacy. Although most outcomes revealed no significant heterogeneity, moderate heterogeneity was noted for any AE outcome. Possible differences among the included studies may have contributed to this heterogeneity, including variations in intervention protocols regarding laterality, as well as differences in baseline age and follow-up duration. These factors likely influenced the heterogeneity, and therefore, the results should be interpreted with caution. Lastly, the retrospective nature of the studies contributes to the risk of recall bias and missing data, which may limit the clinical applicability of these findings. Addressing these limitations in future research will be crucial for enhancing the reliability and relevance of comparative effectiveness studies in this area.

## Author’s conclusion

DBS and MRgFUS both provide meaningful tremor reduction for patients with ET, but their benefits differ in important clinical ways. DBS may offer greater overall improvement in total CRST scores and remains particularly advantageous for patients with bilateral or disabling axial tremor, given its adjustability and established bilateral efficacy. In contrast, MRgFUS represents an effective noninvasive alternative that may be preferable for patients who wish to avoid implanted hardware or who prioritize a single-session, incisionless procedure, while recognizing its higher rates of certain sensory and gait-related AEs.

A major limitation of the current evidence is the lack of long-term data and the near absence of studies evaluating bilateral MRgFUS, which prevents definitive comparison with bilateral DBS. Additionally, variability in outcome definitions across studies limits consistency in reported effects. Future research should prioritize balanced bilateral comparisons, longer follow-up, and standardized outcome measures to more accurately determine the relative benefits and risks of both procedures.

## Supplementary Information

Below is the link to the electronic supplementary material.

Supplementary file1 (DOCX 528 KB)

## Data Availability

Data were publicly available.

## Declarations

We confirm that this manuscript complies with all instructions to authors, that all authors meet the authorship criteria and have approved the final version of the manuscript, and the PRISMA checklist was used.

### Generative AI in scientific writing

We declare that we have not used AI in writing any part of this abstract.

### Previous publication

We declare that this study has not been published at all.
