# Supplementary material for: Deep brain stimulation and magnetic resonance–guided focused ultrasound for essential tremor: a meta-analysis of effectiveness and safety
Source: Neurosurg Rev. 2026 Mar 9;49(1):264. doi: 10.1007/s10143-025-04076-x (PMC12971766; doi:10.1007/s10143-025-04076-x)
Supplement: Supplementary file 1 — Supplementary file1 (DOCX 528 KB) [file 10143_2025_4076_MOESM1_ESM.docx]

**Supplementary Table 2. Detailed Search Strategy for Each Database**

| **Database** | **Search Terms** | **Search Field** | **Search Results** |
| --- | --- | --- | --- |
| **PubMed** | (("Deep brain stimulation" OR DBS) AND ("Focused ultrasound" OR FUS)) **AND** ("Essential tremor" OR "Essential tremors" OR "Kinetic tremor" OR "Kinetic tremors" OR "Familial tremor" OR "Familial tremors"). | All fields | 129 |
| **Scopus** | ALL ((("Deep brain stimulation" OR DBS) AND ("Focused ultrasound" OR FUS)) **AND** ("Essential tremor" OR "Essential tremors" OR "Kinetic tremor" OR "Kinetic tremors" OR "Familial tremor" OR "Familial tremors")) | All fields | 168 |
| **WOS** | (("Deep brain stimulation" OR DBS) AND ("Focused ultrasound" OR FUS)) **AND** ("Essential tremor" OR "Essential tremors" OR "Kinetic tremor" OR "Kinetic tremors" OR "Familial tremor" OR "Familial tremors"). | All fields | 300 |
| **Cochrane CENTRAL** | (("Deep brain stimulation" OR DBS) AND ("Focused ultrasound" OR FUS)) **AND** ("Essential tremor" OR "Essential tremors" OR "Kinetic tremor" OR "Kinetic tremors" OR "Familial tremor" OR "Familial tremors"). | All fields | 11 |

**Supplementary Table 2. Risk of Bias Assessment Using the Newcastle-Ottawa Scale (NOS)**
Evaluation of the methodological quality of the included studies. Four studies scored 8/9 (good quality), while one scored 6/9 (poor quality) due to issues with comparability.

| **Study** | **Selection** | | | | **Comparability** | **Outcome** | | | | **Total** | |
| --- | --- | --- | --- | --- | --- | --- | --- | --- | --- | --- | --- |
|  | **Representativeness of the Exposed Cohort** | **Selection of the Non-Exposed Cohort** | **Ascertainment of Exposure** | **Demonstration that Outcome Was Not Present at Baseline** |  | **Assessment of Outcome** | **Follow-Up Duration** | **Adequacy of Follow-Up** |  | |  |
| Huss 2015 | * | * | * | * | * | * | * | * | 8/9 (Good) | |  |
| Kim 2017 | * | * | * | * | * | * | * | * | 8/9 (Good) | |  |
| Harary 2019 | * | * | * | * | 0 | * | * | * | 6/9 (Poor) | |  |
| Germann 2024 | * | * | * | * | * | * | * | * | 8/9 (Good) | |  |
| Sarica 2025 | * | * | * | * | * | * | * | * | 8/9 (Good) | |  |

The NOS tool reviews each observational study based on nine criteria that are organized into three groups: participant selection, group comparability, and the determination of either the exposure or outcome of interest. Each criterion is rated as either 'yes' or 'no'. Studies that achieve a score between 7 and 9 are classified as high quality, while those scoring between 5 and 6 are regarded as fair quality. Scores that fall between 1 and 4 are identified as poor quality.

**Supplementary Table 3. Adverse Events Reported in Included Studies**
Comprehensive list of all adverse events reported across studies, including events not eligible for meta-analysis due to limited reporting. Events include dysarthria, paresthesia, infection, mental status changes, hemorrhage, and others.

| Adverse events | Studies reported | The incidence rate in the FUS group (Event/Total, (%)) | The incidence rate in the DBS group (Event/Total, (%)) |
| --- | --- | --- | --- |
| Any AE | 3 | 61/121, (50.4%) | 43/74, (58.1%) |
| Gait instability | 5 | **51/192, (26.56%)** | **11/271, (4.05%)** |
| Dysarthria | 4 | 7/169, (4.14%) | 18/252, (7.14%) |
| Dysphagia | 3 | **7/90, (7.77%)** | **3/219, (1.37%)** |
| Paresthesia | 3 | **20/150, (13.33%)** | **5/230, (2.17%)** |
| Mental status change | 2 | 0/71, (0%) | 4/197, (2.03%) |
| Infection | 2 | 0/71, (0%) | 4/197, (2.03%) |
| Hemorrhage | 2 | 0/71, (0%) | 3/197, (1.522%) |
| Headache | 1 | 0/15, (0%) | 0/70, (0%) |
| Nause/vomiting | 1 | 0/15, (0%) | 0/70, (0%) |
| Dizziness | 1 | 0/15, (0%) | 0/70, (0%) |
| Flushed warmth | 1 | 0/15, (0%) | 0/70, (0%) |
| Lead erosion | 1 | 0/15, (0%) | 2/70, (2.8%) |
| Mild facial paresis | 1 | 1/23, (4.34%) | 1/19, (5.26%) |
| Muscle twitching | 1 | 1/23, (4.34%) | 0/19, (0%) |
| Paresis | 1 | 1/56, (1.785%) | 1/127, (0.79%) |
| Dysmetria | 1 | 2/56, (3.57%) | 0/127, (0%) |
| Tinnitus | 1 | 0/56, (0%) | 1/127, (0.79%) |
| Seizure | 1 | 0/56, (0%) | 1/127, (0.79%) |
| Stroke | 1 | 0/56, (0%) | 1/127, (0.79%) |
| Intracranial edema | 1 | 0/56, (0%) | 1/127, (0.79%) |
| Lead reposition | 1 | 0/56, (0%) | 2/127, (1.575%) |
| Lead replacement | 1 | 0/56, (0%) | 3/127, (2.36%) |
| Motor weakness | 1 | 1/19, (5.26%) | 1/22, (4.54%) |
| Dysesthesia | 1 | **5/19, (26.3%)** | **1/22, (4.54%)** |
